# Effect of connexin 43 in LPS/IL-4-induced macrophage M1/M2 polarization: An observational study

**DOI:** 10.1097/MD.0000000000037811

**Published:** 2024-04-12

**Authors:** Pengchen He, Mingxing Dai, Zongpin Li, Xiaoyi Wang, Hongyuan Liu, Yixiao He, Han Jiang

**Affiliations:** a Department of Neurosurgery, Mianyang Central Hospital Affiliated to University of Electronic Science and Technology of China, Mianyang, China; b Department of Rehabilitation Therapy, Mianyang Central Hospital Affiliated to University of Electronic Science and Technology of China, Mianyang, China; c Department of Pathology, Mianyang Central Hospital Affiliated to University of Electronic Science and Technology of China, Mianyang, China.

**Keywords:** connexin 43, inflammation, macrophage, polarization, tumor

## Abstract

Lipopolysaccharide (LPS) and interleukin-4 (IL-4) play important roles in inducing M1 and M2 macrophage polarization. Studies have shown that LPS can promote the polarization of macrophages to M1-type and produce many pro-inflammatory cytokines, while IL-4 can promote the polarization of macrophages to M2-type and produce many anti-inflammatory cytokines. Moreover, Connexin 43 (Cx43) is widely expressed in macrophages and has various regulatory functions. However, whether Cx43 is involved in the regulation of macrophage M1/M2 polarization has not been fully studied. This study examined the role of Cx43 and M2 polarization markers using Western blot, immunofluorescence, flow cytometry. Cx43 overexpression was induced using Cx43 overexpressing lentivirus. The statistical software SPSS 20.0 (IBM Corp.) and GraphPad Prism 8.0 (GraphPad Software, La Jolla, CA, United States) were used to analyze the results. *P* values < .05 were considered to indicate statistically significant differences. Our results showed that LPS promotes the polarization of macrophages to M1-type, which is accompanied by an increase in Cx43 expression from 0 to 24 hours. Moreover, the application of the Cx43-specific blockers Gap19 and Gap26 reduces the expression of macrophage M1-type polarization markers. Thus, the expression of Cx43 increases first, and then, due to the initiation of intracellular autophagy during LPS-induced macrophage M1 polarization. Cx43 is degraded and the expression of Cx43 decreases from 24 hours to 48 hours. IL-4 decreases the expression of Cx43 from 24 hours to 48 hours and promotes the transformation of macrophages to M2-type. The application of Cx43 overexpression lentivirus leads to a reduction in the expression of M2 polarization markers. IL-4-induced M2 polarization of macrophages inhibits cell autophagy, reducing Cx43 degradation and leading to an increase in Cx43 from 24 hours to 48 hours. Thus, Cx43 expression in M2-type polarization experiences a reduction at first and then an increase from 24 hours to 48 hours. The direction of macrophage polarization can be controlled by regulating the expression of Cx43, thus providing a theoretical basis for treating atherosclerosis, tumors, and other diseases associated with macrophage polarization.

## 1. Introduction

Macrophages are one of the most common immune cells in the body, and they play an irreplaceable role in the progression of various diseases.^[1]^ Macrophage polarization can be divided into different phenotypes.^[2]^ Among these, the most studied are M1-type and M2-type macrophages.^[3]^ Lipopolysaccharide (LPS) and Interferon-γ (IFN-γ) can induce macrophage polarization toward M1-type, which secrete inflammatory factors such as TNF-α, IL-6, and IL-1.^[4]^ M1-type macrophages are also characterized by significant increases in CD86 and inducible nitric oxide synthase (iNOS), which play an important role in the pro-inflammatory response, accelerating atherosclerosis and anti-tumor processes.^[5]^

In contrast, M2 polarization can be induced by interleukin-4 (IL-4) and IL-13 and leads to the secretion of anti-inflammatory cytokines. Macrophage M2 polarization plays crucial roles in reducing the inflammatory response and accelerating tumor progression.^[6,7]^ The process of autophagy is affected by M2-type polarization. Indeed, it has been reported that M2-type polarization inhibits the process of autophagy. Besides, the increase of M2-type macrophages favors tumor progression.^[8]^

Gap junction channels or hemichannels composed of connexins are widely expressed in macrophages and are involved in the immune response to various diseases. Gap junctions play a significant role in cell-cell interactions and signal transduction by allowing ions with a molecular weight <1.5 kDa to pass through freely.^[9]^ Connexins are the basic components of gap junctions. The hemi-channel of connexins is made up of 6 hexamers formed by 6 of the same connexins.^[10]^ The connexin hemichannels in 2 closely adjacent cells form the gap junction. Recent studies have shown that Cx30, Cx31, and Cx40 play key roles in the macrophage polarization process.^[11]^ The maturation of airway epithelium cells has also been associated with the down-regulation of Cx26 and connexin 43 (Cx43), while Cx30 has been shown to be involved in astroglial polarization process.

Cx43 is the most widely expressed connexin in macrophages.^[12]^ To explore the effect of Cx43 in immune process, the inhibitor of Cx43 Gap19 and Gap26 is widely used. They were small peptides. Previous studies indicated that Cx43 mimetic peptides Gap26 and Gap27 may protect cerebral ischemic injury. Gap19 is a new specific inhibitor of Cx43 hemichannels.^[13]^ Gap19 blocks HCs but not GJCs and is specific for Cx43.^[14]^

However, the mechanism by which Cx43 acts in M1 and M2 polarization remains unclear and whether Cx43 can influence related diseases by regulating macrophage polarization is also unknown. Recent studies show that formononetin can reduce inflammation and atherosclerosis by influencing macrophage polarization. Whether there are other ways to alter polarization and thus reduce inflammation is not known.^[15]^ Therefore, the main purpose of this paper was to explore whether Cx43 affects M1/M2-type polarization, and if so, what role it plays, with the aim to provide clues for the development of a new treatment for AS and cancer.

## 2. Materials and methods

### 2.1. Cell culture

RAW264.7 macrophages were bought from the Shanghai Institutes for Biological Sciences, Chinese Academy of Sciences. The ethical approval was not necessary, because this experiment only used cells. The cells were cultivated in a culture dish at a density of 3 × 10^5^/mL in a CO_2_ thermostatic incubator at 37°C and 5% CO_2_ in Dulbecco Modified Eagle Media (DMEM; Gibco, Thermo Fisher Scientific, Inc.) with 12% fetal bovine serum (Gibco, Thermo Fisher Scientific, Inc.). The cells were passaged after reaching 70% to 80% confluence.

RAW264.7 macrophages were cultured and treated as follows: Control group, macrophages cultured in DMEM/fetal bovine serum with no treatment; LPS group, cells were treated with 100 ng/mL LPS (Sigma) for 24 hours at 37°C^[16]^; IL-4 group, cells were pre‑treated with 20 ng/mL IL-4 (Protechnol) at 37°C for 24 hours; LPS + Cx43 inhibitor Gap19 or Gap26 group, cells were pre‑treated with 2 × 10^‑5^ mol/l Gap19 or 2 × 10^‑5^ mol/l Gap26 (APExBIO Technology LLC) at 37°C for 30 minutes prior to treatment with 100 ng/mL LPS for 24 hours; IL-4 + Cx43 overexpression lentivirus group, cells were pre‑treated with 20 mol/L Cx43 overexpression lentivirus (Genechem) at 37°C for 30 minutes prior to treatment with 20 ng/mL IL-4 for 24 hours; Macrophages were treated with LPS (100 ng/mL) for 0 hours, 6 hours, 12 hours, 24 hours, or 48 hours, and with IL-4 (20 ng/mL) for 0 hours, 6 hours, 12 hours, 24 hours, or 48 hours, respectively.

### 2.2. Western blot

The protein expression levels of CD86, CD206, iNOS, Arginase-1 (Arg-1), Cx43, Beclin-1, and LC3B were detected by Western blot. Briefly, macrophages were lysed on ice for at least 15 minutes. Total protein was extracted from cells using RIPA lysis buffer (cat. no. R0020; Beijing Solarbio Science and Technology Co., Ltd.) and quantified using the BCA method. Samples with equivalent amounts of protein were separated by SDS-PAGE in 5 × loading buffer. The separated proteins were then transferred onto a PVDF membrane (EMD Millipore) and blocked with 5% skimmed milk in TBS‑0.2% Tween‑20 (TBST; Sangon Biotech, Co., Ltd.) for 2 hours at room temperature. Next, the membranes were incubated with primary mouse or rabbit antibodies specific for the relative proteins at 4°C overnight: Anti‑CD86 (1:1000, ab39075, Abcam, United Kingdom), anti‑Cx43 (1:1000, ab314908, Abcam, United Kingdom), anti‑iNOS (1:1000, ab178945, Abcam, United Kingdom), anti‑CD206 (1:1000, ab64693, Abcam, United Kingdom), anti‑β‑actin (1:1000, ab8226, Abcam, United Kingdom), anti-Beclin-1 (1:1000, ab207612, Abcam, United Kingdom), anti-LC3B (1:1000, ab192890, Abcam, United Kingdom), and anti‑GAPDH (1:1000, ab8245, Abcam, United Kingdom). After primary antibody incubation, the membranes were washed 3 times with TBST (10 minutes/wash) and the secondary antibody (1:10000, PV-6001-3ml, Beijing Zhongshan Jinqiao Biotechnology, Beijing, China) was incubated with the PVDF membrane at room temperature for 2 hours. The membrane was washed 3 times with TBST (10 minutes/wash) before incubating with ECL reagent (GE Healthcare Life Sciences, London, United Kingdom). The collected images were analyzed using ImageJ software (version 1.8.0; National Institutes of Health). All of theses reagents used green nanomaterials.

### 2.3. Immunofluorescence

To generate cell slides, 4 × 10^5^/mL RAW264.7 macrophages were plated into 6-well plates at 37°C for 24 hours with sterile coverslips. Next, the slides were washed with phosphate-buffered saline (PBS; Beijing Solarbio Science & Technology Co., Ltd.) thrice and then incubated with 4% paraformaldehyde for 10 minutes at room temperature. The slides were then subjected to 3 washes with PBS (2–3 minutes/wash), permeabilized with 0.2% Triton X-100 for 3 minutes at room temperature, washed thrice with PBS (3 minutes per wash), and then blocked with 5% bovine serum albumin (BSA; Sigma-Aldrich; Merck KGaA) blocking solution at 37°C for 30 minutes. Subsequently, sections were probed with relevant primary antibodies (anti-Cx43 polyclonal antibody [1:100, Abcam], anti-CD86 polyclonal antibody [1:100, Abcam],^[17]^ and anti-CD206 polyclonal antibody [1:100, Abcam]). Samples were incubated with the primary antibody for 3 hours at 37°C. Samples were washed thrice with PBS (2 minutes/wash) prior to incubation with the fluorescent secondary antibody (1:50; Goat anti-rabbit secondary antibodies; Beijing Zhongshan Jinqiao Biotechnology Co., Ltd.) for 1 hour at 37°C. Following incubation, the secondary antibody solution was discarded and samples were washed 3 times with PBS (3 minutes/wash). Slides were subsequently incubated with propidium iodide (1:1000; Beijing Solarbio Science & Technology Co., Ltd.) at 10°C at room temperature in the dark for 10 minutes before being washed 3 times with PBS (10 minutes/wash). An anti-fluorescence attenuating sealer was then added to the slides and the staining was observed using a Zeiss LSM 510 META laser confocal microscope (magnification, ×630; Carl Zeiss AG, Germany).

### 2.4. Flow cytometry

CD86 (Invitrogen; Thermo Fisher Scientific, Inc.) expression was detected by flow cytometry. Cells (1.5 × 10^6^/well) were cultivated for 24 hours, digested with 0.25% trypsin, centrifuged at 1000 rpm/min for 5 minutes at 37°C, and subsequently retained as a pellet. The supernatant was discarded and each pellet was resuspended with 1 mL PBS, centrifuged at 1000 rpm/min at 37°C for 5 minutes, and retained as a pellet. Using 200 µL PBS to resuspend the pellets in Eppendorf tubes, 2 µL phycoerythrin (PE)‑CD86 antibody (1:100, MHCD8604, Invitrogen; Thermo Fisher Scientific, Inc.) was added to each Eppendorf tube and cultured at 37°C in the dark for 45 to 50 minutes.^[18]^ Subsequently, PBS was added to each tube and the cells were centrifuged at 1000 rpm/min for 5 minutes at 37°C. The supernatant was removed and the pellets were resuspended in 200 µL PBS. Formulated fixative Fixation Concentrate (BD Biosciences) was added to each tube at room temperature for 20 minutes before incubation with a PE‑CD206 antibody (1:100, 12-2069-42, Invitrogen; Thermo Fisher Scientific, Inc.) at 37°C in the dark for 30 minutes. Subsequently, cells were resuspended in 1 mL 1 × permeabilization reagent (BD Biosciences) for 30 minutes at 25°C and centrifuged at 1000 rpm/min for 5 minutes at 37°C. The supernatant was discarded, the cell pellets were resuspended with 200 µL PBS, and CD206 and CD86 expression levels were analyzed by flow cytometry (FACSort; BD Biosciences) with BD CellQuest Pro software (version 2.0, system OS2; Becton, Dickinson and Company).

### 2.5. Statistical analysis

The statistical software SPSS 20.0 (IBM Corp.) and GraphPad Prism 8.0 (GraphPad Software, La Jolla, CA) were used to analyze the results. The values are expressed as means ± standard errors, and one-way analysis of variance (ANOVA) and t-test were used to analyze the differences among groups. *P* values < .05 were considered to indicate statistically significant differences.

## 3. Results

### 3.1. Cx43 is increased in LPS-induced M1-type macrophages and inhibiting Cx43 reduces the expression of M1 polarization markers

**Figure 1.**
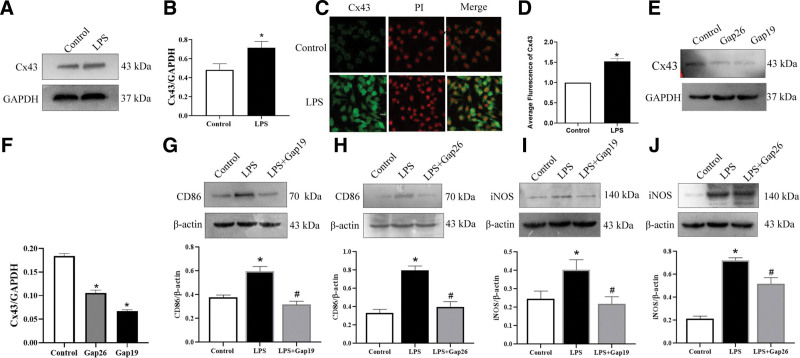
Expression levels of Cx43 in LPS-induced macrophage polarization. (A) Total protein levels of Cx43 in RAW264.7 macrophages in the control and LPS groups. (B) Statistical analysis of Cx43. Data are presented as the mean ± SEM (n = 3). ^*^P < .05 vs control groups. (C) Expression levels of Cx43 in RAW264.7 macrophages as detected by immunofluorescence (n = 3). (D) Statistical analysis of Cx43. Data are presented as the mean ± SEM (n = 3). ^*^P < .05 vs control groups. (E) Total protein levels of Cx43 in RAW264.7 macrophages in the control, Gap26 and Gap19 groups. (F) Statistical analysis of Cx43. Data are presented as the mean ± SEM (n = 3). ^*^P < .05 vs control groups. ^#^*P *< .05 vs LPS groups. (G–H) The total expression levels of CD86 in RAW264.7 macrophages in the control, LPS, LPS + Gap19, and LPS + Gap26 groups (n = 3). (I–J) The total expression level of iNOS in RAW264.7 macrophages in the control, LPS, LPS + Gap19, and LPS + Gap26 groups (n = 3). Data are presented as the mean ± SEM (n = 3). ^*^P < .05 vs control groups. ^#^*P *< .05 vs LPS groups. Cx43 = connexin 43, iNOS = inducible nitric oxide synthase, LPS = lipopolysaccharide, PI = propidium iodide.

To investigate the level of Cx43 in LPS-induced M1 polarization, a Western blot was used to detect the expression levels of Cx43 in RAW264.7 macrophages. Compared with the control groups, the levels of Cx43 expression were significantly increased following LPS treatment for 24 hours (Fig. 1A and B). Furthermore, the localization of Cx43 in RAW264.7 macrophages was determined using an immunofluorescence assay. The results showed that Cx43 was expressed in RAW264.7 macrophages. Compared with the control groups, the protein expression levels of Cx43 in macrophages were consistent with the results obtained from Western blotting (Fig. 1C and D).

To investigate the role of Cx43 in macrophage M1-type polarization, RAW264.7 macrophages were pretreated with Gap19 or Gap26 for 30 minutes before being treated with LPS for 24 hours. Western blot was used to detect the effect of Gap19 and Gap26 in reducing Cx43 expression. The results showed that Cx43 levels were significantly reduced in Gap19 and Gap26 groups (Fig. 1E and F). Western blotting analysis demonstrated that CD86 and iNOS expression levels in the LPS groups were significantly increased compared with the control groups, and were significantly decreased in the LPS + Gap19 and LPS + Gap26 groups compared with the LPS groups (Fig. 1G–J).

**Figure 2.**
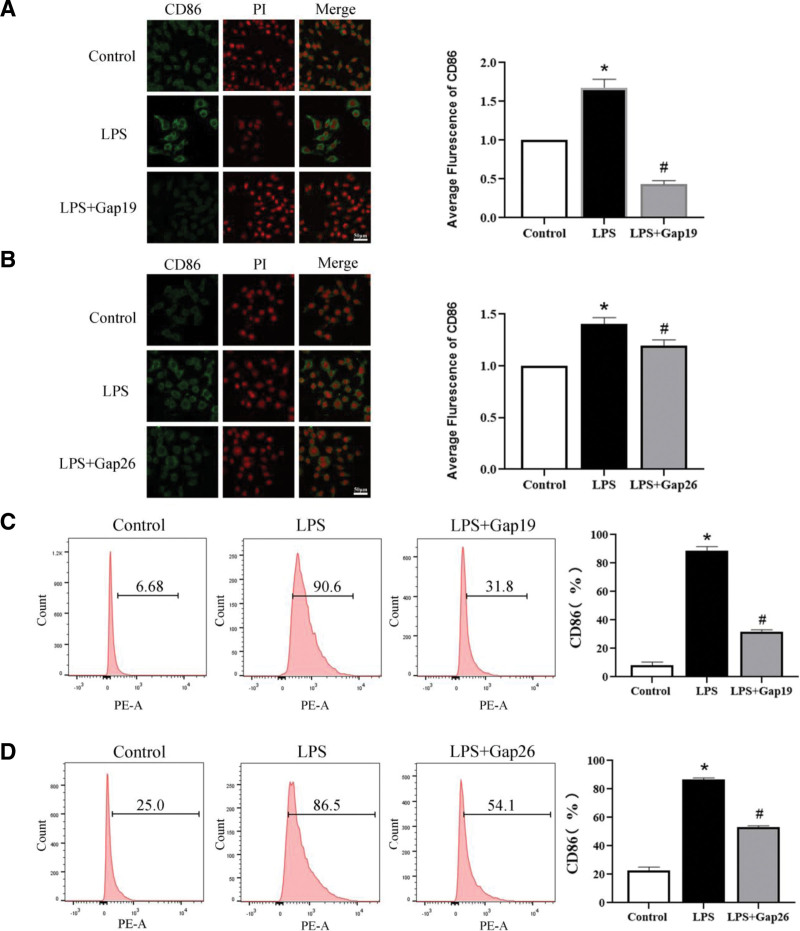
Expression of M1-type polarization markers with Cx43 inhibitors. (A–B) The location and expression levels of CD86 in RAW264.7 macrophages pretreated with Gap19 and Gap26 in the control, LPS, LPS + Gap19, and LPS + Gap26 groups (n = 3). (C–D) Flow cytometry was used to detect the expression frequency of CD86 in RAW264.7 macrophages pretreated with Gap19 and Gap26 (n = 3). Data are presented as the mean ± SEM (n = 3). ^*^P < .05 vs control groups; ^#^*P *< .05 vs LPS groups. Cx43 = connexin 43, LPS = lipopolysaccharide, PE = phycoerythrin.

Furthermore, the localization and expression levels of CD86 in macrophages were analyzed using an immunofluorescence assay. The results indicated that CD86 was expressed in the cell membrane and nucleus. Compared with the control groups, the protein expression levels of CD86 in the LPS groups were higher than those in the control groups, and were decreased in the LPS + Gap19 and LPS + Gap26 groups compared with the LPS groups (Fig. 2A and B). These results were consistent with those obtained from Western blotting. Additionally, the frequency of CD86 expression in macrophages was detected by flow cytometry. The levels of CD86 were significantly higher in the LPS groups than in the control groups, and this increase was prevented by pretreatment with Gap19 and Gap26 (Fig. 2C and D). These results suggest that blocking Cx43 can decrease the expression of macrophage M1 polarization markers.

### 3.2. M1-type polarization induces autophagy in macrophages and decreases Cx43 expression at 48 hours

**Figure 3.**
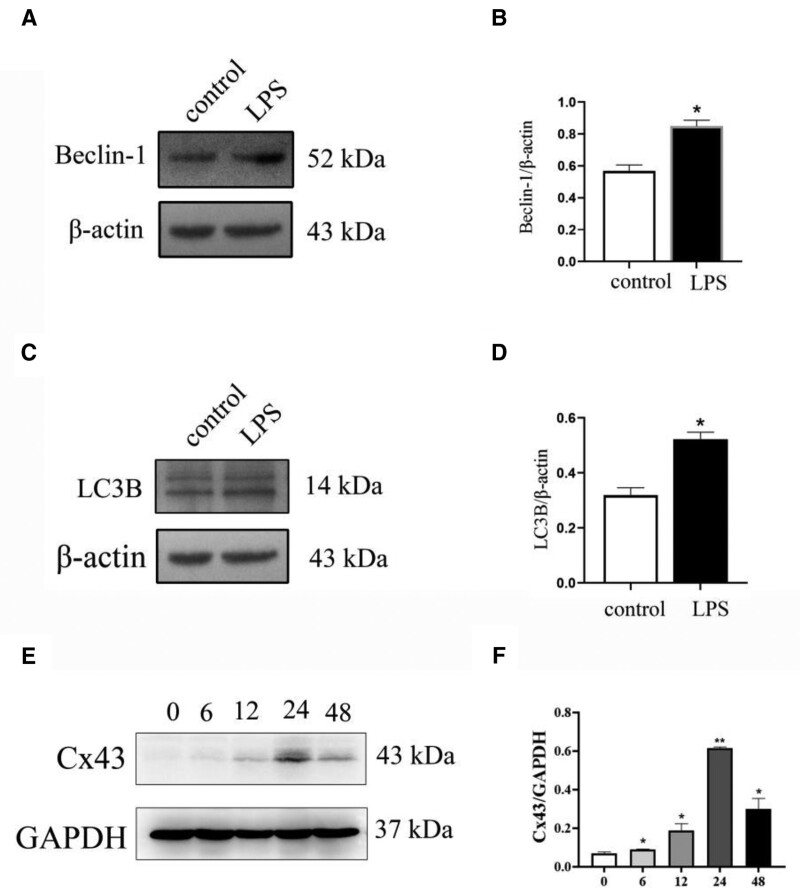
Expression of autophagy-related markers Beclin-1 and LC3B following 48 h LPS treatment. (A) Western blotting was used to detect the expression levels of Beclin-1 in macrophages pretreated with LPS (n = 3). (B) Statistical analysis of Beclin-1. (C) Western blotting was used to detect the expression levels of LC3B in macrophages pretreated with LPS (n = 3). (D) Statistical analysis of LC3B. (E) Total protein expression levels of Cx43 in RAW264.7 macrophages treated with 100 ng/mL LPS at different time points (0, 6, 12, 24, and 48 h). (F) Statistical analysis of Cx43. Data are presented as the mean ± SEM (n = 3). ^*^P < .05 vs control groups, ^**^P < .01 vs control groups. Cx43 = connexin 43, LPS = lipopolysaccharide.

To investigate the role of autophagy in macrophage M1-type polarization, RAW264.7 macrophages were pretreated with LPS for 48 hours. Western blotting analysis showed that Beclin-1 and LC3B expression levels in the LPS groups were significantly increased compared with the control groups (Fig. 3A–D). To further characterize the role of Cx43 in autophagy, Cx43 protein expression was detected using Western blot following treatment with 100 ng/mL LPS for 0, 6, 12, 24, and 48 hours. The protein expression levels of Cx43 first increased, before reaching a maximum at 24 hours and then decreasing (Fig. 3E and F).

### 3.3. Cx43 is decreased following IL-4-induced M2 polarization from 0 to 24 hours

**Figure 4.**
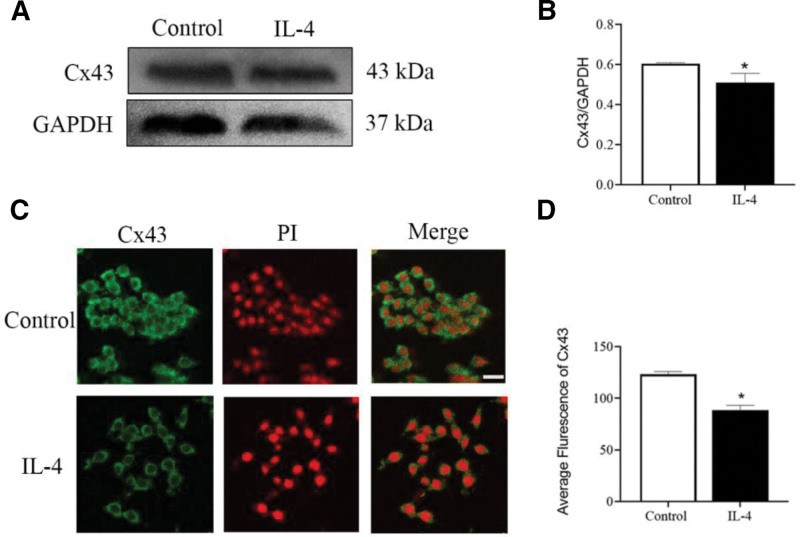
Expression of Cx43 in IL-4-induced M2-type polarization. (A) Total expression levels of Cx43 in RAW264.7 macrophages in the control and IL-4 groups (n = 3). (B) Statistical analysis of Cx43. (C) The expression levels of Cx43 in RAW264.7 macrophages in the control and IL-4 groups were detected by immunofluorescence (n = 3). (D) Statistical analysis of Cx43. Data are presented as the mean ± SEM (n = 3). ^*^P < .05 vs control groups. Cx43 = connexin 43, IL-4 = interleukin-4.

To investigate the effect of Cx43 in IL-4-induced M2 polarization, a Western blot was used to detect the expression levels of Cx43 in RAW264.7 macrophages. Compared with the control groups, the levels of Cx43 expression were significantly decreased following IL-4 treatment for 24 hours (Fig. 4A and B). The localization of Cx43 in RAW264.7 macrophages was next determined using an immunofluorescence assay. The results showed that Cx43 was expressed in the membrane and nucleus of macrophages. Compared with the control groups, the protein expression levels of Cx43 in the IL-4 group were lower than those in control groups, which were consistent with the results obtained from Western blot (Fig. 4C and D).

### 3.4. Overexpression of Cx43 reduces the expression of macrophage M2 polarization markers

**Figure 5.**
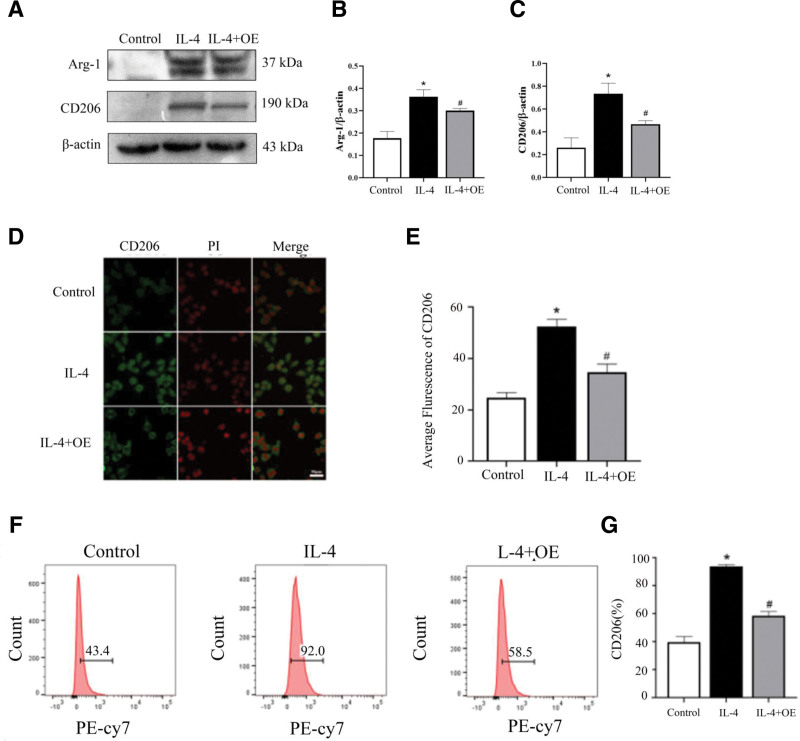
Expression of M2-type polarization markers with Cx43 agonist. (A) Western blotting was used to detect the expression levels of Arg-1 and CD206 in macrophages pretreated with OE. (B) Western blotting was used to detect the expression levels of Arg-1 and CD206 in macrophages treated with OE. Expression of M2-type polarization markers with Cx43 agonist. (D–E) The location and expression levels of CD206 in RAW264.7 macrophages pretreated with OE in the control, IL-4, IL-4 + OE groups (n = 3). (F–G) Flow cytometric analysis was used to detect the expression frequency of CD206 in RAW264.7 macrophages pretreated with OE (n = 3). Data are presented as the mean ± SEM (n = 3). ^*^P < .05 vs control groups; ^#^*P *< .05 vs. Arg-1 = arginase-1, Cx43 = connexin 43, IL-4 = interleukin-4, OE = overexpress.

To investigate the role of Cx43 in macrophage M2 polarization, RAW264.7 macrophages were pretreated with Cx43 overexpression lentiviral (OE) for 30 minutes before being treated with IL-4 for 24 hours. Western blotting analysis demonstrated that CD206 and Arg-1 expression levels in the IL-4 group were significantly increased compared with those in the control groups. However, CD206 and Arg-1 were significantly decreased in the IL-4 + OE groups compared with the IL-4 groups (Fig. 5A–C). The localization and expression levels of CD206 in macrophages were analyzed using an immunofluorescence assay. The results indicated that CD206 was expressed in the membrane and nucleus of macrophages. Compared with the control groups, the protein expression of CD206 was higher in the IL-4 groups than in the control groups, and was decreased in the IL-4 + OE groups compared with the IL-4 groups. These results were consistent with those obtained from Western blotting (Fig. 5D and E). The expression of CD206 in macrophages was also detected using flow cytometry. The results showed that the levels of CD206 were significantly higher in the IL-4 groups compared with those in the control groups, and this increase was prevented by pretreatment with OE (Fig. 5F and G). These results suggest that overexpressing Cx43 can decrease the expression of macrophage M2 polarization markers.

### 3.5. M2-type polarization reduces autophagy in macrophages at 48 hours, and Cx43 protein expression was first decreased and then increased by IL-4 in RAW264.7 macrophages

**Figure 6.**
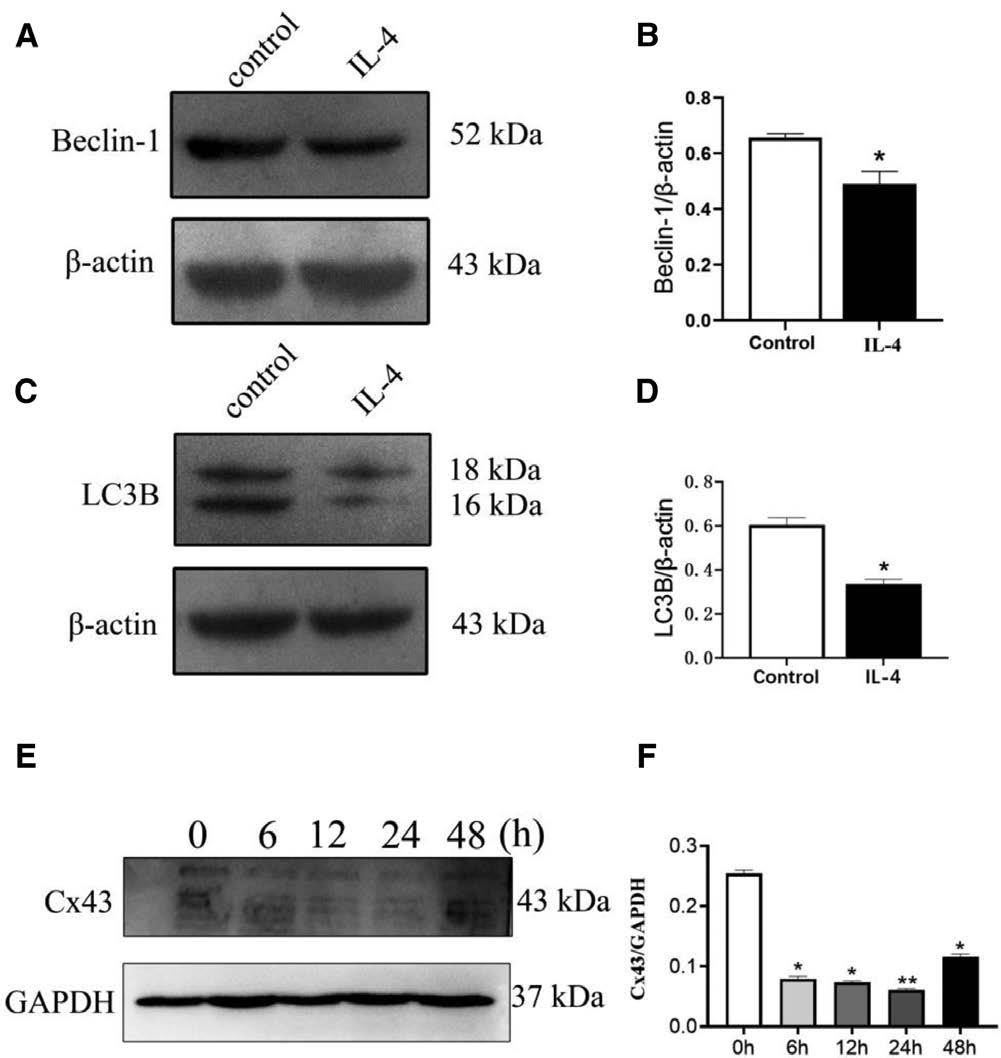
Expression of autophagy-related markers with the intervention of IL-4 at 48 h. (A) Western blotting was used to detect the expression levels of Beclin-1 in macrophages pretreated with IL-4 (n = 3). (B) Statistical analysis of Beclin-1. Data are presented as the mean ± SEM (n = 3). ^*^P < .05 vs control groups (C) Western blotting was used to detect the expression levels of LC3B in macrophages pretreated with IL-4 (n = 3). (D) Statistical analysis of LC3B. Data are presented as the mean ± SEM (n = 3). ^*^P < .05 vs control groups. (E) Western blotting was used to detect the protein expression levels of Cx43 in macrophages treated with 20 ng/mL IL-4 at 0, 6, 12, 24, and 48 h. (F) Statistical analysis of Cx43. Data are shown as the mean ± SEM (n = 3). ^*^P < .05 vs control groups, ^**^P < .01 vs control groups. Cx43 = connexin 43, IL-4 = interleukin-4.

To investigate the role of autophagy in macrophage M2-type polarization, RAW264.7 macrophages were pretreated with IL-4 for 48 hours. Western blotting analysis demonstrated that Beclin-1 and LC3B expression levels in the IL-4 group were significantly lower than those in the control groups at 48 hours (Fig. 6A–D). Treatment of macrophages with 20 ng/mL IL-4 for 0, 6, 12, 24, and 48 hours led to a first decrease and then an increase in Cx43 protein expression. (Fig. 6).

## 4. Discussion

The polarization of macrophages is easily affected by external environmental factors. Under the stimulation of different environmental factors, macrophages differentiated into M1 or M2 subtypes.^[19]^ Different subtypes of cells may play different roles, such as pro-inflammatory or anti-inflammatory effects. In the present study, Western blot, immunofluorescence and flow cytometry showed that 24-h LPS treatment of macrophages led to increases in the expression of CD86 and iNOS compared with the control groups (Figs. 1 and 2). These results corroborate the findings of numerous previous studies. In contrast, M2-type macrophages are mainly induced by IL-4 and secrete anti-inflammatory factors such as CD206, Arg-1, and interleukin-10.^[20]^ M2-type macrophages regulate the function of immune system, inhibit inflammation, and promote tumor formation.^[21]^ In this study, Western blot, immunofluorescence and flow cytometry demonstrated that 24-h IL-4 treatment of macrophages resulted in an increase in the expression of M2-type polarization markers CD206 and Arg-1 compared with the control groups (Fig. 5). These results are consistent with the findings of numerous previous studies.

Macrophages possess various connecting channels.^[22]^ Gap junction channels are composed of connexin proteins, including Cx32, Cx37, Cx40, and Cx43, all of which are widely expressed in immune cells.^[23]^ Cx43 is the most widely expressed on various immune cells and plays an important role in immune response.^[24]^ Studies have shown that macrophages express Cx43, and that Cx43 is upregulated following stimulation with LPS and IFN-γ.^[25]^ Moreover, the ability of macrophages to cross the blood-brain barrier is decreased after intervention with the nonspecific gap blocker 18α-glycyrrhizic acid.^[26]^ This result suggests that Cx43 can regulate the function of macrophages.^[27]^ In our work, we detected the expression of Cx43 in macrophage M1- and M2-type polarization and found that Cx43 was widely expressed (Figs. 1 and 4).

As specific blockers of Cx43, Gap19 and Gap26 are involved in the regulation of cell function. The main function of Gap19 is to regulate the domain of the Cx43 CL pH receptor and block the Cx43 semi-channel.^[28]^ In the myocardial ischemia-reperfusion model, Gap19 has been shown to decrease the expression of Cx43 in mitochondria, thereby reducing the size of myocardial infarction. It has also been found that Gap19 can block Cx43 and reduce apoptosis,^[29]^ indicating that Gap19 may reduce Cx43 expression and affect the physiological function of cells and the progression of disease. Gap26, another specific blocker of Cx43, is a synthetic peptide derived from the extracellular ring of Cx43.^[30]^ Studies have shown that Gap26 can protect the heart from ischemic damage in rats, while treatment with Gap26 after ischemia can reduce the damage of heart tissue,^[31]^ suggesting that Gap26 can affect disease process by blocking Cx43. Researchers have found that Gap-134, a Cx43 activator, prevents age-related development of ventricular fibrosis in Scn5a^±^ mice. These results indicate that the regulation of Cx43 can affect the disease process. We found that Gap19, Gap26, and Cx43-OE are involved in the regulation of macrophage polarization by blocking or overexpressing Cx43. Western blot, flow cytometry, and immunofluorescence were used to detect the expression of M1- and M2-type polarization markers in macrophages to explore whether Cx43 could be involved in regulating the M1/M2-type polarization of macrophages. The results indicated that, compared with the control group, LPS intervention could increase the protein expression of Cx43 in macrophages. Blocking Cx43 reduced the expression of M1-type polarization markers (Figs. 1 and 2), IL-4 treatment decreased the protein expression of Cx43 in macrophages, and Cx43 overexpression lentivirus reduced the expression of M2-type polarization markers (Fig. 4). These results suggest that Cx43 is involved in the regulation of M1- and M2-type macrophage polarization.

The polarization process of macrophages is closely associated with autophagy. Studies have shown that in the process of acute liver failure induced by D-galactosamine and LPS, autophagy significantly inhibits the expression of inflammatory factors in the liver and significantly deteriorates liver tissue injury.^[32]^ Other studies have shown that the percentage of autophagosomes increased significantly in LPS-induced macrophages. Indeed, Xu et al found that LPS induces the formation of autophagosomes under the regulation of the TICAM1/TRIF-dependent TLR4 pathway in macrophages.^[33]^ IFN-γ promotes autophagy by promoting autophagosome maturation,^[34,35]^ however, some Th2 cytokines, such as IL-4 and IL-13, inhibit famine-induced autophagy through the Akt/mTOR pathway and IFN-γ-induced autophagy through the STAT6 pathway. Therefore, macrophage M1-type polarization increases macrophage autophagy in 24 hours (Fig. 3A–D). During the process of macrophage M2-type polarization, the autophagy level of macrophages decreased gradually (Fig. 6A–D).

Cx43 is regulated by autophagy and gap junctions are degraded by autophagy under physiological conditions. Previous studies have found that autophagy leads to the degradation of Cx43 during ischemia,^[36]^ and gene silencing of autophagy-related genes leads to the accumulation of Cx43 and decreases the co-localization of LC3B and Cx43.^[37]^ Relevant studies have also shown that during the middle part of M1-type macrophage polarization, autophagy is enhanced, leading to increased degradation of Cx43.^[38]^Our results showed that autophagy was increased at 48 hours which led to a decrease of Cx43 at 48 hours (Fig. 3). Thus, Cx43 showed an increase from 0 to 24 hours and decreased in 48 hours.

It has been reported that during macrophage M2-type polarization, the autophagy level of macrophages gradually decreased.^[35]^ The results of Figure 6 are consistent with the previous reports. The level of autophagy decreased in 48 hours and Cx43 expression increased due to the decreased expression of autophagy in 48 hours (Fig. 6). Therefore, Cx43 experienced a decrease from 0 to 24 hours and an increase in 48 hours due to the autophagy.

This research demonstrated that Cx43 might be a potential target to regulate M1/M2 type polarization. Macrophage polarization is associated with the development and progression of several diseases, including cancer and AS. Recent studies have shown that M2 macrophage-derived exosomal miR-26b-5p regulates macrophage polarization and chondrocyte hypertrophy by targeting toll-like receptor 3 and COL10A1 to alleviate osteoarthritis.^[39]^ Besides, studies have found that Cinobufagin upregulated MME and inactivated FAK/STAT3 signaling to regulate macrophage reprogramming and inhibit triple-negative breast cancer. Thus, the regulation of macrophage phenotype through Cx43 may be related to some diseases which suggests some research is needed to be done in the future. Other studies have shown that deficiency or inhibition of IDO-1 alleviated hepatocytes ferroptosis and M1 polarization induced by hepatic I/R injury, while also enhancing M2 polarization and promoting efferocytosis in macrophages. Furthermore, depletion of macrophages attenuated ferroptosis in hepatocytes induced by hepatic I/R injury. Our work represents a solid alternative to existing therapeutics.^[40]^ Quercetin-induced suppression of transient receptor potential vanilloid subtype 1 leads to a delay in osteoarthritis progression by shifting the macrophage polarization from M1 to M2 subtypes via modulation of the P2X7/NLRP3 pathway.^[41]^ By regulating Cx43 expression, our work provides an alternative method to osteoarthritis.^[40]^

## 5. Conclusion

**Figure 7.**
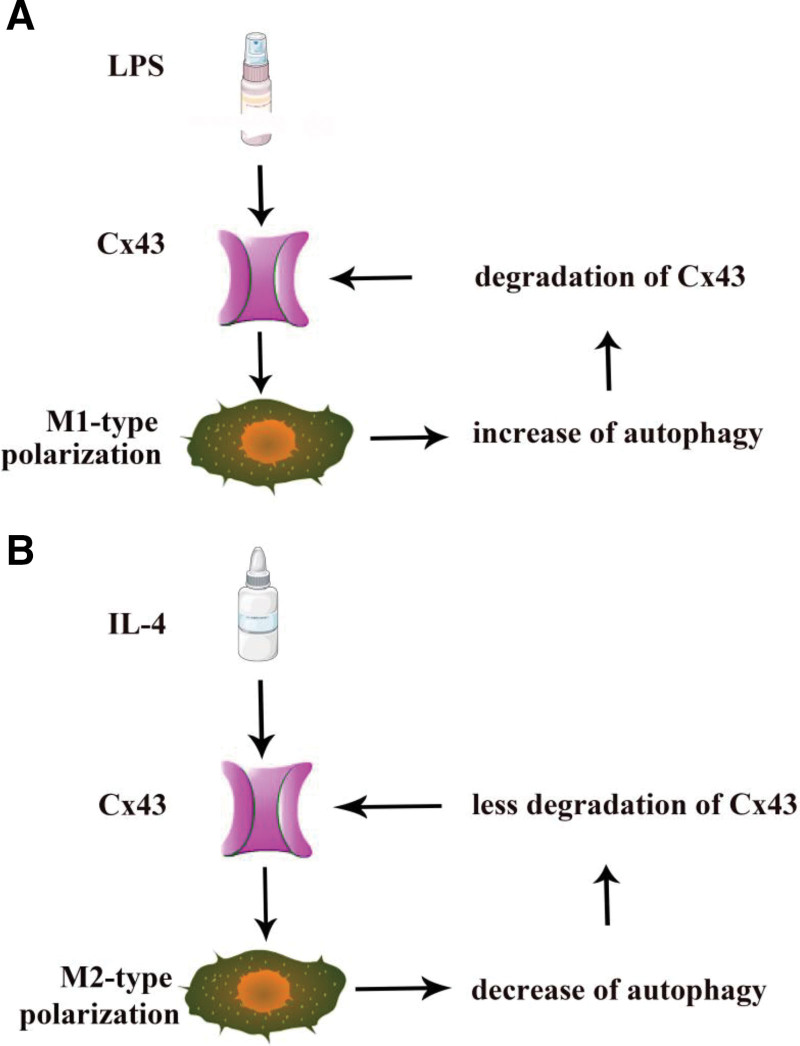
Diagram showing the effect of Cx43 during M1/M2 polarization. (A) Effect of Cx43 during M1-type polarization and autophagy. (B) Effect of Cx43 during M2-type polarization and autophagy. Cx43 = connexin 43.

In conclusion, during LPS-induced M1-type macrophage polarization, Cx43 was first increased, followed by the induction of autophagy, which led to Cx43 degradation at 48 hours (Fig. 7A). During M2-type polarization, Cx43 was first decreased under the induction of IL-4 at 24 hours. Then, autophagy was inhibited, leading to an in Cx43 from 24h to 48 hours (Fig. 7B).

The expression of Cx43 can be regulated—for example, increasing or reducing the expression of Cx43 to modulate macrophage M1/M2 polarization. This may provide new ideas to ultimately allow the regulation of diseases associated with macrophage polarization and provide novel treatments for some diseases, such as AS and cancer.

## Author contributions

**Conceptualization:** Han Jiang, Zongping Li, Xiaoyi Wang, Hongyuan Liu, Yixiao He.

**Data curation:** Pengchen He, Zongping Li.

**Formal analysis:** Mingxing Dai, Xiaoyi Wang, Hongyuan Liu.

**Funding acquisition:** Han Jiang, Hongyuan Liu, Yixiao He.

**Investigation:** Han Jiang.

**Methodology:** Pengchen He, Mingxing Dai.

**Project administration:** Mingxing Dai.

**Resources:** Pengchen He, Mingxing Dai.

**Supervision:** Yixiao He.

**Validation:** Yixiao He.

**Visualization:** Han Jiang, Yixiao He.

**Writing – original draft:** Han Jiang.

**Writing – review & editing:** Han Jiang.

## References

[1] Yang S Yuan H Hao Y. Macrophage polarization in atherosclerosis. Clin Chim Acta. 2020;501:142–6. 31730809 10.1016/j.cca.2019.10.034

[2] Dieli-Conwright CM Parmentier JH Sami N. Adipose tissue inflammation in breast cancer survivors: effects of a 16-week combined aerobic and resistance exercise training intervention. Breast Cancer Res Treat. 2018;168:147–57. 29168064 10.1007/s10549-017-4576-y PMC6233989

[3] Nakagawa M Karim MR Izawa T. Immunophenotypical characterization of M1/M2 macrophages and lymphocytes in cisplatin-induced rat progressive renal fibrosis. Cells. 2021;10:257. 33525592 10.3390/cells10020257 PMC7911194

[4] Baran A Mehmet FB Cumali K. Investigation of antimicrobial and cytotoxic properties and specification of silver nanoparticles (AgNPs) derived from Cicer arietinum L. Green leaf extract. Front Bioeng Biotechnol. 2022;10:855136. 35330628 10.3389/fbioe.2022.855136 PMC8940290

[5] Oishi Y Manabe I. Macrophages in inflammation, repair and regeneration. Int Immunol. 2018;30:511–28. 30165385 10.1093/intimm/dxy054

[6] Chen YN Hu MR Wang L. Macrophage M1/M2 polarization. Eur J Pharm. 2020;877:173090. 10.1016/j.ejphar.2020.173090 32234529

[7] Alvarez MM Liu JC Santiago GT. Delivery strategies to control inflammatory response: modulating M1-M2 polarization in tissue engineering applications. J Control Release. 2016;240:349–63. 26778695 10.1016/j.jconrel.2016.01.026 PMC4945478

[8] Tu DY Dou J Wang MK. M2 macrophages contribute to cell proliferation and migration of breast cancer. Cell Biol Int. 2021;45:831–8. 33325089 10.1002/cbin.11528

[9] Sarrouilhe D Dejean C Mesnil M. Connexin43- and pannexin-based channels in neuroinflammation and cerebral neuropathies. Front Mol Neurosci. 2017;10:320. 29066951 10.3389/fnmol.2017.00320 PMC5641369

[10] Ahmadian E Eftekhari A Samiei M. The role and therapeutic potential of connexins, pannexins and their channels in Parkinson’s disease. Cell Signal. 2019;58:111–8. 30877035 10.1016/j.cellsig.2019.03.010

[11] Zhou Q Wang YY Lu ZS. Cx43 acts as a mitochondrial calcium regulator that promotes obesity by inducing the polarization of macrophages in adipose tissue. Cell Signal. 2023;105:110606. 36681290 10.1016/j.cellsig.2023.110606

[12] Rodjakovic D Salm L Beldi G. Function of connexin-43 in macrophages. Int J Mol Sci. 2021;22:1412. 33573367 10.3390/ijms22031412 PMC7866802

[13] Abudara V Bechberger J Freitas-Andrade M. The connexin43 mimetic peptide Gap19 inhibits hemichannels without altering gap junctional communication in astrocytes. Front Cell Neurosci. 2014;8:306. 25374505 10.3389/fncel.2014.00306 PMC4204617

[14] Wang N De Bock M Decrock E. Connexin targeting peptides as inhibitors of voltage- and intracellular Ca2+-triggered Cx43 hemichannel opening. Neuropharmacology. 2013b;75:506–16. 24007825 10.1016/j.neuropharm.2013.08.021

[15] He Y Cai YD Wei DL. Elucidating the mechanisms of formononetin in modulating atherosclerotic plaque formation in ApoE-/- mice. BMC Cardiovasc Disord. 2024;24:121. 38388385 10.1186/s12872-024-03774-6 PMC10882812

[16] Di Battista AP Rhind SG Shiu M. Whole blood stimulation provides preliminary evidence of altered immune function following SRC. BMC Immunol. 2024;25:6. 38218771 10.1186/s12865-023-00595-8 PMC10788016

[17] Buzzella A Mazzini G Vicini R. A preliminary study of an alternative method for evaluating skin sensitizing potential of chemicals. Int J Cosmet Sci. 2019;41:257–64. 30993720 10.1111/ics.12530

[18] Zhang Y Park YS Kim IB. A distinct microglial cell population expressing both CD86 and CD206 constitutes a dominant type and executes phagocytosis in two mouse models of retinal degeneration. Int J Mol Sci. 2023;24:14236. 37762541 10.3390/ijms241814236 PMC10532260

[19] Ernestina A Tantuoyir MM Zheng R. Exploring the polarization of M1 and M2 macrophages in the context of skin diseases. Mol Biol Rep. 2024;51:269. 38302766 10.1007/s11033-023-09014-y

[20] Zhou DD Ji L Chen GY. TSPO modulates IL-4-induced microglia/macrophage M2 polarization via PPAR-γ pathway. J Mol Neurosci. 2020;70:542–9. 31879837 10.1007/s12031-019-01454-1

[21] Seyedizade SS Afshari K Bayat S. Current status of M1 and M2 macrophages pathway as drug targets for inflammatory bowel disease. Arch Immunol Ther Exp. 2020;68:1–24. 10.1007/s00005-020-00576-4 32239308

[22] Cacialli P Mahony CB Petzold T. A connexin/ifi30 pathway bridges HSCs with their niche to dampen oxidative stress. Nat Commun. 2012;12:4484. 10.1038/s41467-021-24831-0 PMC8302694 34301940

[23] Beyer EC Berthoud VM. Gap junction gene and protein families: connexins, innexins, and pannexins. Biochim Biophys Acta Biomembr. 2018;1860:5–8. 28559187 10.1016/j.bbamem.2017.05.016 PMC5704981

[24] Kameritsch P Pogoda K. The role of connexin 43 and pannexin 1 during acute inflammation. Front Physiol. 2020;11:594097. 33192611 10.3389/fphys.2020.594097 PMC7658380

[25] Liu LQ Yan MJ Yang R. Adiponectin attenuates lipopolysaccharide-induced apoptosis by regulating the Cx43/PI3K/AKT pathway. Front Pharmacol. 2021;12:644225. 34084134 10.3389/fphar.2021.644225 PMC8167433

[26] Kimura K Orita T Morishige N. Role of the JNK signaling pathway in downregulation of connexin43 by TNF-α in human corneal fibroblasts. Curr Eye Res. 2013;38:926–32. 23768164 10.3109/02713683.2013.798419

[27] Tishchenko A Azorín DD Vidal-Brime L. Cx43 and associated cell signaling pathways regulate tunneling nanotubes in breast cancer cells. Cancers (Basel). 2020;12:2798. 33003486 10.3390/cancers12102798 PMC7601615

[28] Wei S Cassara C Lin X. Calcium-calmodulin gating of a pH-insensitive isoform of connexin43 gap junctions. Biochem J. 2019;476:1137–48. 30910801 10.1042/BCJ20180912 PMC12404149

[29] Ma JW Ji DD Li QQ. Inhibition of connexin 43 attenuates oxidative stress and apoptosis in human umbilical vein endothelial cells. BMC Pulm Med. 2020;20:19. 31964358 10.1186/s12890-019-1036-y PMC6975083

[30] Gao RJ Zhang AM Jia QH. The promoting role of Cx43 on the proliferation and migration of arterial smooth muscle cells for angiotensin II-dependent hypertension. Pulm Pharmacol Ther. 2021;70:102072. 34428599 10.1016/j.pupt.2021.102072

[31] Chen B Yang L Chen J. Inhibition of connexin43 hemichannels with Gap19 protects cerebral ischemia/reperfusion injury via the JAK2/STAT3 pathway in mice. Brain Res Bull. 2019;146:124–35. 30593877 10.1016/j.brainresbull.2018.12.009

[32] Mohar I Brempelis KJ Murray SA. Isolation of non-parenchymal cells from the mouse liver. Methods Mol Biol. 2015;1325:3–17. 26450375 10.1007/978-1-4939-2815-6_1

[33] Xu Y Jagannath C Liu XD. Toll-like receptor 4 is a sensor for autophagy associated with innate immunity. Immunity. 2007;27:135–44. 17658277 10.1016/j.immuni.2007.05.022 PMC2680670

[34] Fang C Weng T Hu S. IFN-γ-induced ER stress impairs autophagy and triggers apoptosis in lung cancer cells. Oncoimmunology. 2021;10:1962591. 34408924 10.1080/2162402X.2021.1962591 PMC8366549

[35] Singh SB Davis AS Taylor GA. Human IRGM induces autophagy to eliminate intracellular mycobacteria. Science. 5792;313:1438–41. 16888103 10.1126/science.1129577

[36] Wang GY Bi YG Liu XD. Autophagy was involved in the protective effect of metformin on hyperglycemia-induced cardiomyocyte apoptosis and connexin43 downregulation in H9c2 cells. Int J Med Sci. 2017;14:698–704. 28824303 10.7150/ijms.19800 PMC5562122

[37] Catarino S Ribeiro-Rodrigues TM Ferreira RS. A conserved LIR motif in connexins mediates ubiquitin-independent binding to LC3/GABARAP proteins. Cells. 2020;9:902. 32272685 10.3390/cells9040902 PMC7226732

[38] Li C Shi L Peng C. Lead-induced cardiomyocytes apoptosis by inhibiting gap junction intercellular communication via autophagy activation. Chem Biol Interact. 2021;337:109331. 33242459 10.1016/j.cbi.2020.109331

[39] Qian YF Chu GL Zhang L. M2 macrophage-derived exosomal miR-26b-5p regulates macrophage polarization and chondrocyte hypertrophy by targeting TLR3 and COL10A1 to alleviate osteoarthritis. J Nanobiotechnol. 2024;22:72. 10.1186/s12951-024-02336-4 PMC10877765 38374072

[40] Ma SY Li JJ Ye HX. Indoleamine 2, 3-dioxygenase 1 activation in macrophage exacerbates hepatic ischemia-reperfusion injury by triggering hepatocyte ferroptosis. Int Immunopharmacol. 2024;130:111692. 38382261 10.1016/j.intimp.2024.111692

[41] Li WJ He HB Min D. Quercetin as a promising intervention for rat osteoarthritis by decreasing M1-polarized macrophages via blocking the TRPV1-mediated P2X7/NLRP3 signaling pathway. Phytother Res. 2024;2:1–17. 10.1002/ptr.8158 38372204

